# PTK6 regulates growth and survival of endocrine therapy-resistant ER+ breast cancer cells

**DOI:** 10.1038/s41523-017-0047-1

**Published:** 2017-11-17

**Authors:** Koichi Ito, Sun Hee Park, Igor Katsyv, Weijia Zhang, Carmine De Angelis, Rachel Schiff, Hanna Y. Irie

**Affiliations:** 1 Division of Hematology and Medical Oncology, Department of Medicine, Icahn School of Medicine at Mount Sinai, 1468 Madison Ave, New York, NY USA; 2 Department of Genetics and Genomic Sciences, Icahn School of Medicine at Mount Sinai, New York, NY USA; 3 Division of Nephrology, Department of Medicine, Icahn School of Medicine at Mount Sinai, New York, NY USA; 4 Lester & Sue Smith Breast Center, Dan L. Duncan Comprehensive Cancer Center, Baylor College of Medicine, Houston, TX USA; 5 Department of Medicine, Baylor College of Medicine, Houston, TX USA; 6 Department of Molecular and Cellular Biology, Baylor College of Medicine, Houston, TX USA; 7 Department of Oncological Sciences, Tisch Cancer Institute, Icahn School of Medicine at Mount Sinai, 1468 Madison Ave, New York, NY USA

## Abstract

The non-receptor tyrosine kinase, PTK6/BRK, is highly expressed in multiple tumor types, including prostate, ovarian, and breast cancers, and regulates oncogenic phenotypes such as proliferation, migration, and survival. PTK6 inhibition also overcomes targeted therapy resistance of HER2+ breast cancer. Although PTK6 is highly expressed in ER+ Luminal breast cancers, the role of PTK6 in this subtype has not been elucidated. In this study, we investigated the functions of PTK6 in ER+ Luminal breast cancer cells, including those that are relatively resistant to estrogen deprivation or targeted endocrine therapies used in the treatment of ER+ cancers. Enhanced expression of PTK6 in ER+ breast cancer cells enhances growth of ER+ breast cancer cells, including tamoxifen-treated cells. Downregulation of PTK6 in ER+ breast cancer cells, including those resistant to tamoxifen, fulvestrant, and estrogen deprivation, induces apoptosis, as evidenced by increased levels of cleaved PARP, and an increase in the AnnexinV+ population. PTK6 downregulation impairs growth of these cells in 3D Matrigel^TM^ cultures, and virtually abrogates primary tumor growth of both tamoxifen-sensitive and resistant MCF-7 xenografts. Finally, we show that p38 MAPK activation is critical for PTK6 downregulation-induced apoptosis, a mechanism that we previously reported for survival of HER2+ breast cancer cells, highlighting conserved mechanisms of survival regulation by PTK6 across breast cancer subtypes. In conclusion, our studies elucidate critical functions of PTK6 in ER+ Luminal breast cancers and support PTK6 as an attractive therapeutic target for ER+ breast cancers.

## Therapeutics: enzyme provides drug target for ER+ breast cancer

Drugs that target a tumor-promoting enzyme called protein tyrosine kinase 6 (PTK6) could help treat hormone-receptor positive breast cancer. A team led by Hanna Irie from the Icahn School of Medicine at Mount Sinai in New York, NY, USA, investigated the role of PTK6, also known as breast tumor kinase, in breast cancer cells that grow in response to the hormone estrogen. They boosted the enzyme’s expression in estrogen receptor-expressing breast cancer cells and saw enhanced growth. Conversely, downregulating PTK6 levels led to cell death, including in tumor cells that were resistant to tamoxifen  and other therapies commonly used to treat estrogen-receptor positive breast cancer. The researchers showed that this effect was dependent on the activation of a signaling pathway previously found to be important in another subtype of breast cancer, highlighting a conserved mechanism of cell survival regulation by PTK6.

## Introduction

Approximately 70% of all diagnosed breast cancers express estrogen receptor (ER) and/or progesterone receptor (PR) and are stimulated to grow in the presence of estrogen. Therapies that target estrogen synthesis or ER function, such as aromatase inhibitors and selective estrogen receptor modulators, have been used successfully to treat patients diagnosed with ER+ breast cancer with improvements in survival (reviewed in ref. 1). However, patients with some ER+ cancers, particularly Luminal B tumors, re-present with recurrent disease or metastases, sometimes several years after completing initial treatment. Therefore, there is a clear need to enhance treatment of ER+ breast cancers and identify novel approaches to treating endocrine therapy-resistant cancers.

The sensitivity of ER+ breast cancer cells to endocrine therapies may be modulated by several distinct mechanisms. For example, increased expression of growth factor receptors such as HER2, IGFR, and EGFR, as well as activation of MAPK/ERK, PI3K/AKT, and SRC signaling, can contribute to primary resistance to tamoxifen treatment.^1–9^ Activation of stress-induced MAP kinases such as JNK and P38 were also reported to be associated with tamoxifen resistance.^10–13^ Finally, dysregulation of cell cycle regulators that inactivate the G1/S checkpoint, such as cyclin D1 overexpression, p16 loss or CDK4/6 amplification, may also contribute to endocrine therapy resistance.^14,15^

Here, we report on the role of protein tyrosine kinase 6 (PTK6) in survival and growth of ER+ Luminal breast cancer cells, including those resistant to standard endocrine therapies used in the treatment of patients with ER+ breast cancers. PTK6 is a non-receptor tyrosine kinase that is amplified or highly expressed in a variety of human cancers, including prostate, ovarian, breast, colon, lung, and head and neck cancers (reviewed in refs. 16, 17). Enhanced expression in cancer cell lines of these tissue types promotes proliferation, survival, migration, and metastases.^17–21^ PTK6 copy number gain or amplification represents one mechanism by which PTK6 expression is increased in tumor cells; however, PTK6 protein expression in cancer is also regulated by transcriptional and post-transcriptional mechanisms, including HIF-1α-stimulated transcript expression and HSP90-mediated protein degradation.^22,23^

Higher PTK6 transcript expression in patient tumors is associated with adverse outcomes.^19^ In fact, increased expression of PTK6 in MCF-10A breast epithelial cells was sufficient to confer resistance to the growth inhibitory effects of treatment with lapatinib, a clinically used small molecule inhibitor of HER1/2 kinases.^24^ Furthermore, we reported that PTK6 downregulation impaired the growth of several lapatinib-resistant HER2+ breast cancer cell lines and induced apoptosis by enhancing Bim expression.^25^

Given the growing evidence for a critical role for PTK6 in breast cancer cell survival, we sought to determine the functional role of PTK6 in ER+ Luminal breast cancer cells. Our results suggest that PTK6 may be an attractive candidate therapeutic target to inhibit growth of ER+ breast cancer cells, including endocrine therapy-resistant cells.

## Results

### PTK6 expression is associated with poor survival outcomes promotes growth of ER+ breast cancer cells basally and under estrogen-deprived conditions

**Fig. 1:**
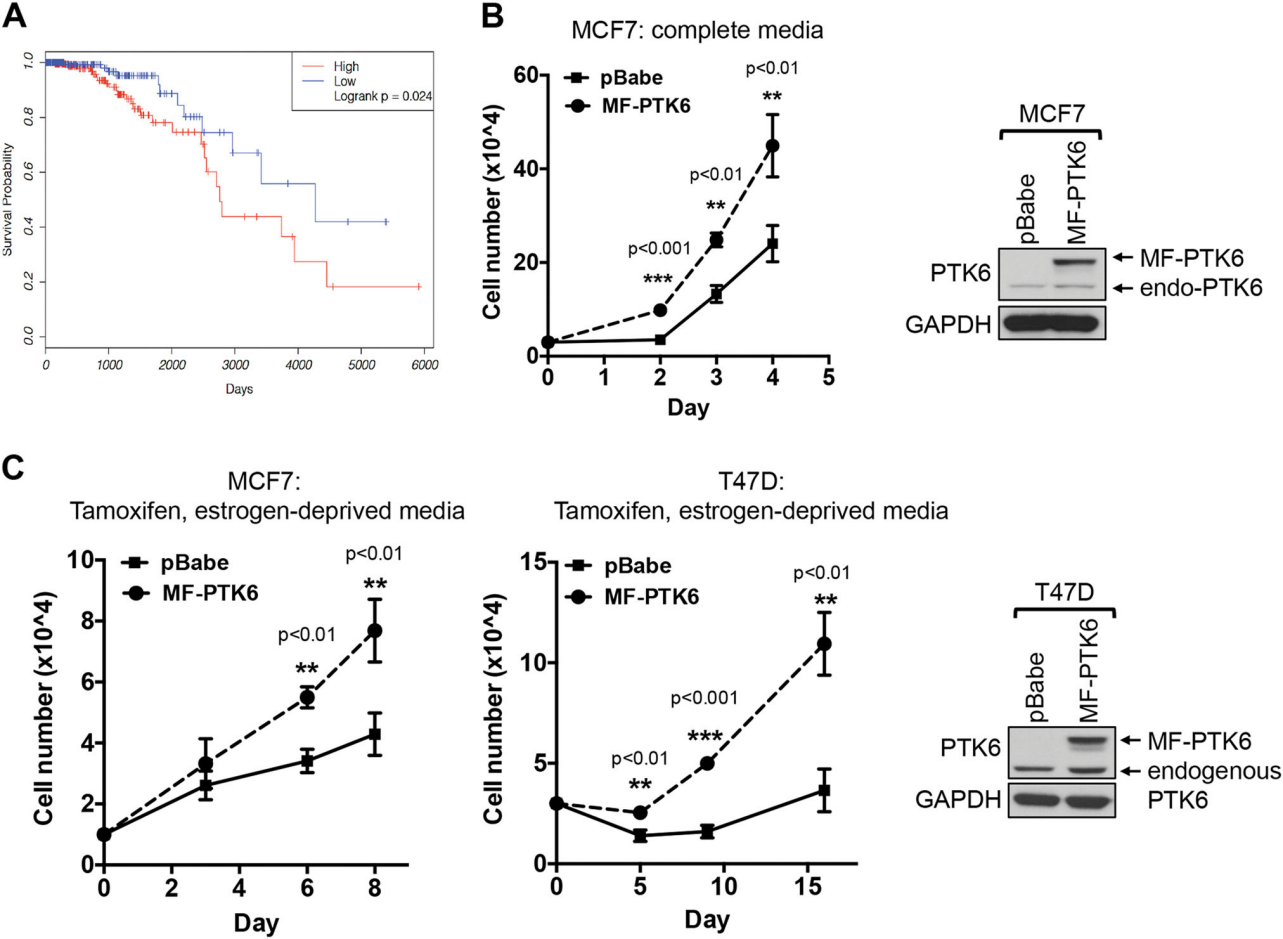
PTK6 expression is associated with poor prognosis and promotes growth of ER+ breast cancer cells. **a** Higher relative PTK6 transcript levels are associated with reduced survival in patients with ER+ breast cancer in the TCGA RNAseqV2 cohort. “High” and “low” PTK6-expressing groups of patients are defined as the top (*n* = 207) and bottom (*n* = 207) tertiles, respectively, based on log2-transformed PTK6 expression. **b**, **c** MCF-7 or T47D cells stably expressing either vector control or activated, membrane-targeted PTK6 (MF-PTK6) were cultured in complete media or in the presence of 4-OHT (1 μM for MCF-7 or 100 nM for T47D cells). Cells were re-fed with 4-OHT containing media every 2–3 days and the cell numbers were counted. Figures are representative of three independent experiments. Error bars are standard error of the mean (s.e.m.). All the western blot images were processed in parallel

PTK6 expression has prognostic significance for patients with ER+ breast cancers. In our analysis of ER+ breast cancer patients in The Cancer Genome Atlas,^26,27^ higher relative expression of PTK6 transcript is associated with poor overall survival (Fig. 1a). This is consistent with our previous analysis of two other publically available microarray data sets with long-term patient outcome data,^28,29^ whereby higher levels of PTK6 expression within the ER+ subtype were associated with adverse patient outcomes.^19^ The prognostic significance of PTK6 is independent of Luminal A/B subtyping; there was no consistently significant difference in level of PTK6 transcript expression between Luminal A and B tumors (Supplemental Fig. 1). Based on these in silico results, we next investigated the role of PTK6 in promoting the growth of ER+ breast cancer cells, including those that are resistant to endocrine therapies.

To determine the effect of enhanced PTK6 on growth of ER+ breast cancer cells, we overexpressed active PTK6 (MF-PTK6) in ER+ MCF-7 and T47D breast cancer cell lines, which are relatively sensitive to tamoxifen and undergo growth arrest in response to tamoxifen treatment.^30^ Expression of MF-PTK6 in MCF-7 and T47D results in active PTK6, as indicated by enhanced levels of autophosphorylated PTK6^31^ (Fig. 1b, c). PTK6 overexpression was sufficient to enhance growth of ER+ breast cancer cells in full growth medium culture conditions (Fig. 1b), as well as in the presence of tamoxifen and estrogen-deprived cultures conditions (e.g., charcoal-stripped serum, phenol red-free medium) (Fig. 1c). These results support the ability of enhanced PTK6 expression to promote ER+ breast cancer cell growth basally and under estrogen-deprived conditions.

### PTK6 downregulation impairs 3D growth and induces apoptosis of endocrine therapy-sensitive and resistant ER+ breast tumor cells

**Fig. 2:**
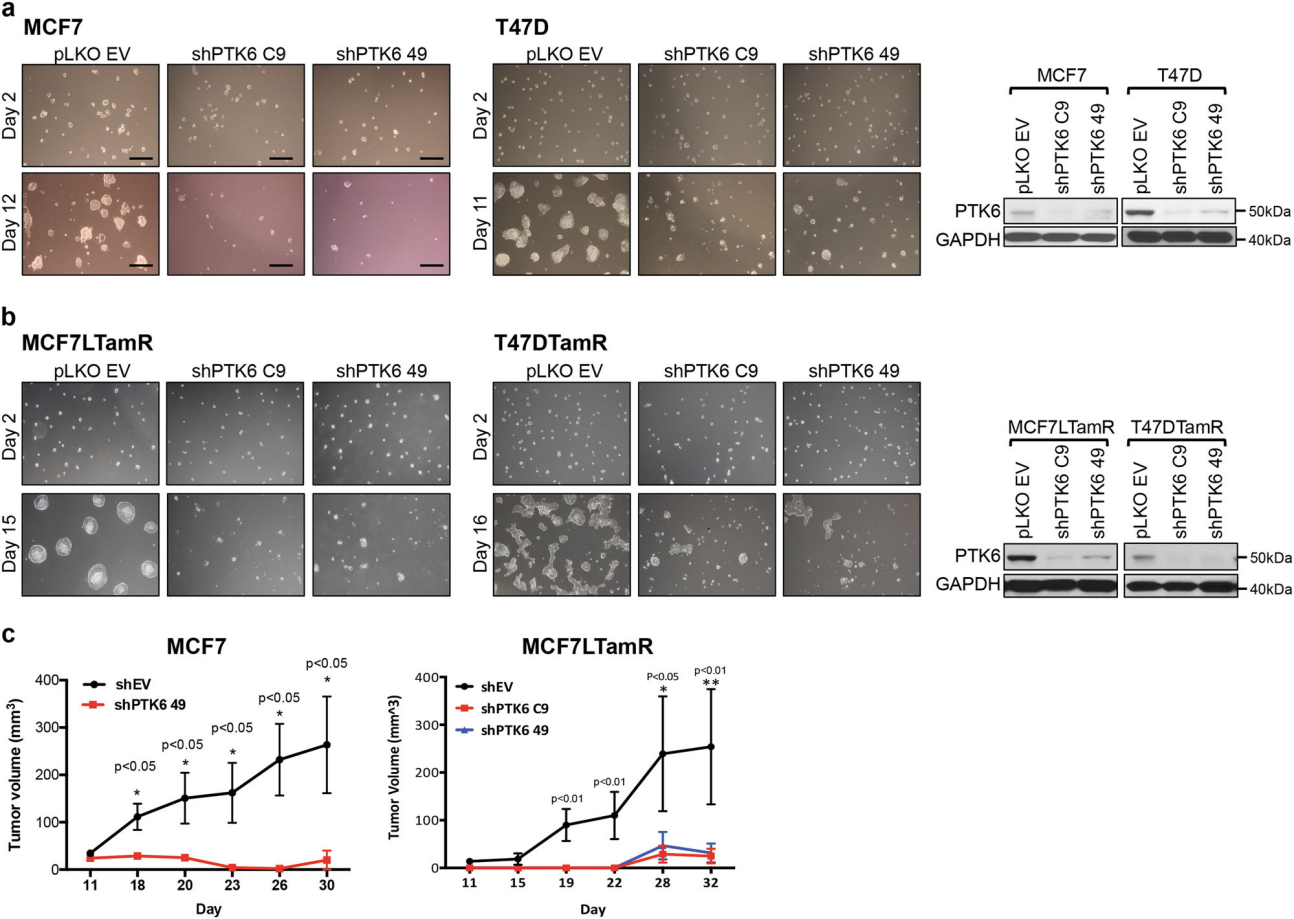
PTK6 downregulation suppresses growth and induces death of ER+ breast cancer cells. **a** MCF-7 or T47D cells or **b** MCF-7L-TamR or T47D-TamR infected with control shRNA (EV) virus or either of two PTK6 shRNA (C9 and 49) viruses were seeded onto chamber slides coated with 3D Matrigel^TM^. Cells were re-fed with complete media every 3 days and growth was monitored. Scale bars represent 30 μM. Lysates from the PTK6 shRNA infected cells (MCF-7 at 96 h or T47D at 120 h post infection) were probed with the indicated antibodies. All experiments were performed three times. All the western blot images were processed in parallel. **c** MCF-7 or MCF-7LTamR cells expressing control or PTK6 shRNAs (C9 or 49) were injected subcutaneously into the right flank of 6-week-old female nude mice implanted with 17β-estradiol pellet (*n* = 5/group). Tumor size was measured every 3–4 days

To further elucidate the functional role of PTK6 in ER+ breast cancer cells, we used two independent shRNA vectors (C9 or 49) to downregulate expression of PTK6 in ER+ cells. PTK6 shRNA expression suppressed growth of parental MCF-7 and T47D cells in 3D Matrigel^TM^ cultures (Fig. 2a). PTK6 downregulation also inhibited 3D growth of ER+ breast cancer cells made resistant to tamoxifen treatment by continuous drug exposure (MCF-7L-TamR and T47D-TamR cells)^32,33^ (Fig. 2b). Finally, PTK6 downregulation inhibited growth of tamoxifen-sensitive (parental) and tamoxifen-resistant ER+ MCF-7L primary tumor xenografts (Fig. 2c).

**Fig. 3:**
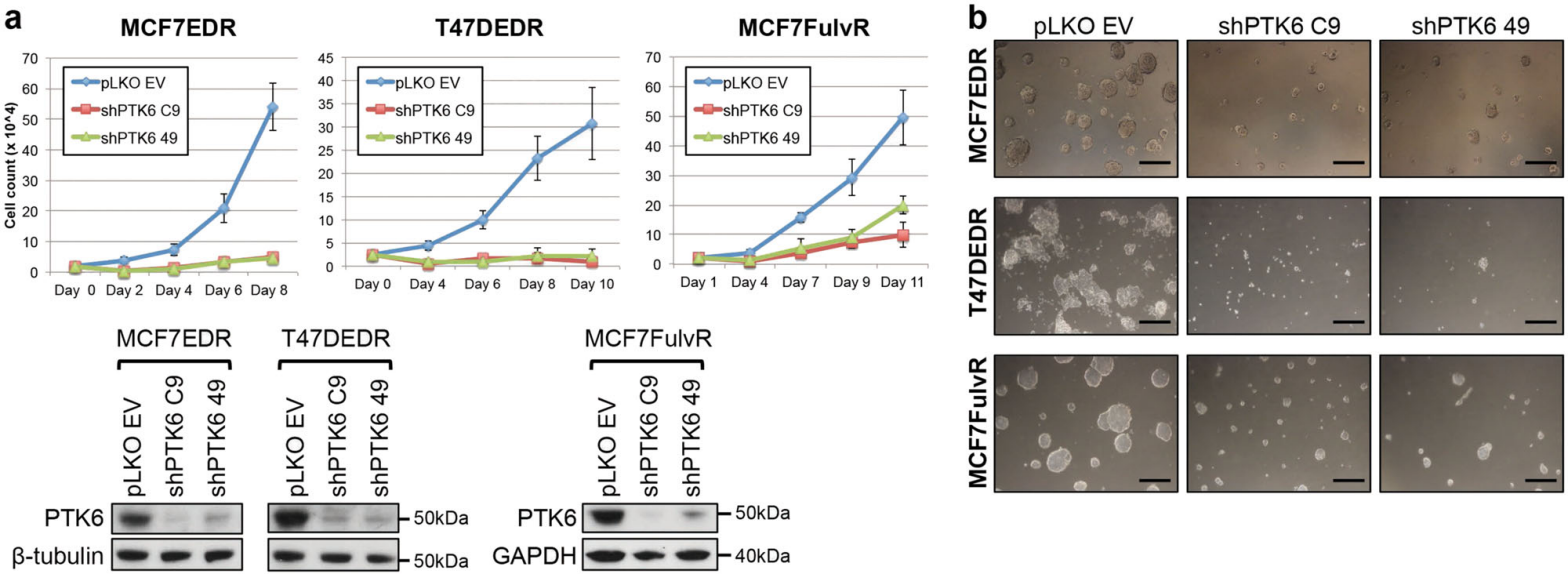
PTK6 downregulation suppresses growth of endocrine therapy-sensitive and resistant ER+ breast cancer cells. MCF-7-EDR, T47D-EDR, or MCF-7-FulvR cells infected with control shRNA (EV) virus or either of two PTK6 shRNA (C9 and 49) viruses were seeded in monolayer cultures (**a**) or 3D Matrigel^TM^ cultures (**b**). Cells were re-fed with fresh growth media every 3–4 days. The cells in the 3D cultures were visualized on day 10. Lysates from the PTK6 shRNA-infected cells were probed with the indicated antibodies. Scale bar: 30 μm. All experiments were performed three times. All the western blot images were processed in parallel

**Fig. 4:**
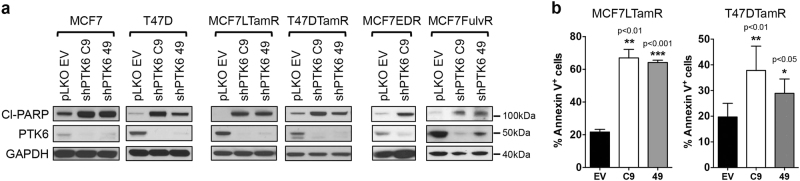
PTK6 downregulation induces apoptosis of endocrine therapy-sensitive and resistant ER+ breast cancer cells. **a** Cells expressing control or PTK6 shRNAs (C9 or 49) were lysed at 96 h (MCF-7, MCF-7L-TamR, MCF-7-EDR, MCF7-FulvR) or 120 h (T47D, T47D-TamR) following infection with shRNA lentivirus. Lysates were probed with antibodies to cleaved PARP, PTK6, and GAPDH. Experiments were performed three times. All the western blot images were processed in parallel. **b** MCF-7L-TamR or T47D-TamR cells expressing control or PTK6 shRNAs (C9 or 49) were stained with AnnexinV and PI, and analyzed by flow cytometry. Experiments were performed five times

PTK6 is critical not only for the growth of ER+ breast cancer cells that are resistant to tamoxifen, but also for the growth of ER+ breast cancer cells made resistant to long-term estrogen deprivation (MCF-7-EDR; T47D-EDR) or to the selective ER degrader fulvestrant (MCF-7-FulvR). In all these models, PTK6 downregulation inhibited cell growth in both monolayer and 3D culture assays (Fig. 3a, b). The impaired growth observed in endocrine therapy-sensitive and resistant ER+ breast cancer models is in part due to cell death. Indeed, PTK6 downregulation resulted in enhanced levels of cleaved-PARP and an increase in the AnnexinV-positive population, consistent with enhanced apoptosis (Fig. 4a, b). These data support a role for PTK6 in survival of endocrine therapy-resistant ER+ breast cancer cells.

Based on these results, we conclude that PTK6 downregulation induces apoptosis and inhibits the in vitro and in vivo growth of ER+ breast cancer cells, including those resistant to endocrine therapies currently used in clinical practice.

### PTK6 inhibition induces cell death by activating p38 MAPK

**Fig. 5:**
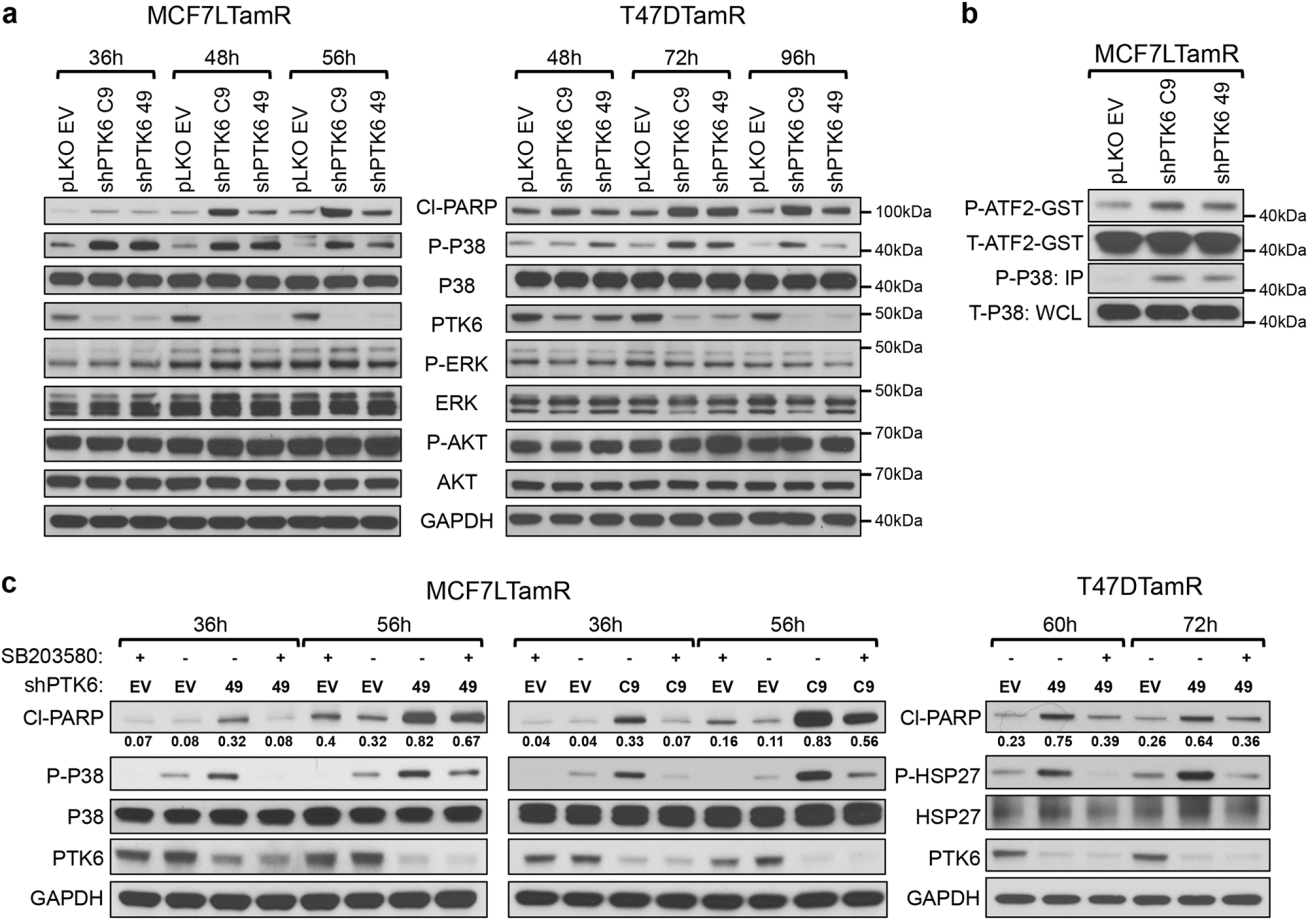
PTK6 downregulation activates p38 MAPK. **a** Lysates from MCF-7L-TamR or T47D-TamR cells expressing control or PTK6 shRNAs (C9 and 49) were probed with the indicated antibodies. **b** MCF-7L-TamR cells were lysed 48 h after shRNA lentiviral infection. Active, phosphorylated p38 was immunoprecipitated and in vitro kinase assay was performed in the presence of ATP (200 μM) and GST-ATF2 substrate (1 μg). The kinase reaction mixture was probed with antibodies to phospho-ATF2, ATF2, phospho-p38 and P38. **c** Cells expressing either control or PTK6 shRNAs (49 or C9) were treated with DMSO or SB203580 (5 μM). Cells were lysed at 36 and 56 h (MCF-7L-TamR) or 60 and 72 h (T47D-TamR) after shRNA lentiviral infection. Lysates were probed with the indicated antibodies. All experiments were performed three times. All the western blot images were processed in parallel

We sought to determine the mechanisms responsible for apoptosis induced by PTK6 downregulation in ER+ breast cancer cells. We examined the status of PI3K/AKT and ERK/MAPK signaling, as these are major survival signaling pathways in ER+ breast cancer cells. We did not observe any changes in levels of AKT or ERK activation with PTK6 downregulation (Fig. 5a). We previously identified p38 MAPK activation as a mechanism responsible for PTK6 shRNA-induced apoptosis of HER2-targeted therapy-resistant breast cancer cells.^25^ To determine whether this mechanism of survival regulation by PTK6 might be conserved in ER+ breast cancer cells, we assessed p38 activity in tamoxifen-resistant ER+ breast cancer cells expressing PTK6 shRNA. PTK6 downregulation enhanced activation of p38 MAPK, as reflected by increased levels of phospho-p38 (Thr180/Tyr182), which temporally preceded the increased levels of cleaved PARP in MCF-7L-TamR and T47D-TamR cells (Fig. 5a). We also assessed p38/MAPK activity toward ATF2, a known p38 substrate using in vitro kinase assays; in PTK6 shRNA-expressing cells, phosphorylation of ATF2 was increased (Fig. 5b).

To determine if p38 activity is required for PTK6 shRNA-induced apoptosis of ER+ breast cancer cells, we treated PTK6 shRNA-expressing cells with SB203580, a p38 MAPK inhibitor. Treatment with SB203580 suppressed PTK6 downregulation-associated apoptosis of MCF-7L-TamR cells and T47D-TamR cells (Fig. 5c). These results support p38 MAPK activation as a mechanism responsible for apoptosis induced by PTK6 downregulation in ER+ breast cancer cells, and p38 regulation is a conserved mechanism by which PTK6 regulates survival of breast cancer cells of distinct subtypes.

## Discussion

The majority of breast cancers diagnosed express ER and/or PR, making them candidates for treatment with endocrine therapies such as tamoxifen and aromatase inhibitors. While many patients with ER and/or PR-positive tumors are successfully treated with these therapies, some experience recurrences even several years after their initial diagnosis, indicative of intrinsic or acquired resistance (reviewed in ref. 1). While chemotherapy remains a treatment option, there has been much recent excitement about targeted therapies that overcome endocrine therapy resistance or enhance sensitivity to these therapies. For example, CDK4/6 and mTOR inhibitors are approved targeted therapies that have helped prolong survival for many patients with advanced ER+ breast cancers that may have recurred despite endocrine therapy.^34^ Therefore, identifying novel candidates that can be targeted to suppress growth of endocrine therapy-resistant cancer cells are necessary to continue to improve outcomes for patients.

In this study, we show that PTK6 expression promotes growth of ER+ breast cancer cells even in the presence of tamoxifen, and PTK6 inhibition induces apoptosis of ER+ breast cancer cells, including those resistant to estrogen deprivation and ER-targeted therapies, including the ER degrader fulvestrant. Therefore, PTK6 is an attractive candidate therapeutic target for ER+ breast cancer.

Multiple mechanisms contribute to the growth of endocrine therapy-resistant breast cancers, including activation of PI3K/AKT and ERK/MAPK signaling, and activation of growth factor receptor signaling such as HER2, EGFR, and IGF-1R.^1,6,7^ In our present study, PTK6 downregulation in tamoxifen-resistant ER+ breast cancer cells does not alter levels of activated PI3K/AKT, ERK or growth factor receptors, including HER2, EGFR, and IGF-1R (data not shown). PTK6 shRNA expression increases p38 activity, as shown by increased levels of phospho-p38 and in vitro kinase activity against ATF2, and p38 activation is required for full apoptosis induction in PTK6-downregulated cells. This requirement for p38 MAPK activation appears to be conserved between ER+ and HER2+ breast cancer cells; we previously showed that p38 activation is required for apoptosis of lapatinib-resistant HER2+ breast cancer cells in which PTK6 expression was downregulated.^25^

p38 MAPK may play both pro-tumorigenic or anti-tumorigenic roles depending on the specific activating stimuli. Although active, phosphorylated p38 is detected in multiple tumor types (breast, prostate, bladder, and lung), including in the context of endocrine resistance,^4,12^ studies of genetically engineered mouse models and other tumor cells support p38 as a negative regulator of cell cycle progression, as well as a mediator of p53-induced apoptosis.^35^ p38 MAPK regulates apoptosis and autophagy, both basally and in response to stress-inducing stimuli, including chemotherapeutic agents and targeted therapies.^36–39^ Several commonly used chemotherapeutic agents such as cyclophosphamide and oxaliplatin induce apoptosis of cancer cells in vitro by activating p38 MAPK.^40^ In contrast, increased p38 MAPK activity has also been associated with tamoxifen resistance via direct phosphorylation of ERα, thereby increasing its transcriptional activity.^41^ Furthermore, p38 MAPK activation contributes to TGFβ-triggered epithelial-mesenchymal transition,^42^ a process linked to increased tumor cell invasion, dissemination, and chemotherapy resistance. Clearly the cellular context and specific stimuli (acute or chronic) leading to various p38 isoform activation will ultimately determine cell death or survival.

P38 is a reported substrate of PTK6 that is critical for proliferation and Heregulin-induced migration of breast cancer cells.^43^ In these studies, PTK6 downregulation inhibited EGF or Heregulin-induced phosphorylation of p38 MAPK. Interestingly, in contrast to our studies, there was no observed effect of PTK6 shRNA expression on baseline levels of phospho-p38 in the breast cancer cells (T47D) used in these studies. These differences may stem from differences in potencies of PTK6 shRNA vectors and/or the time frames examined; due to apoptosis induced by PTK6 downregulation in our studies, we are not able to generate long-term (2+ week) cultures of PTK6 shRNA-expressing T47D cells, as described and utilized by Ostrander et al.^43^ Longer-term cultures may reflect compensatory changes in response to PTK6 downregulation which could also contribute to differences.

It is possible that PTK6 downregulation induces metabolic, oxidative or inflammatory stress in ER+ breast cancer cells, thereby activating p38 MAPK. Consistent with a hypothesized role for PTK6 in stress signaling, PTK6 was reported to protect cells from autophagic cell death in suspension cultures.^18^ The specific mechanisms by which PTK6 downregulation activate p38 MAPK are the focus of ongoing investigation.

Our current study clearly highlights a critical role for PTK6 in the growth and survival of ER+ breast cancer cells, including those resistant to endocrine therapies. Given the recent development of multiple PTK6 small molecule inhibitors, our ongoing studies will assess the efficacy of PTK6 inhibitor treatment, alone or in combination with endocrine therapy, with respect to suppression of ER+ breast cancer cell growth. These studies could facilitate translation of PTK6 inhibition as an effective therapeutic strategy for ER+ breast cancers resistant to standard endocrine therapies.

## Materials and methods

### Antibodies and reagents

Cleaved-PARP (#9541), phospho-p38 (Thr180/Tyr182, #4511), p38 (#8690), phospho-Erk1/2 (#4370), phospho-Akt (S473; #4060), GAPDH (#2118), and ATF2 (#9226) antibodies were purchased from Cell Signaling Technology. BRK (PTK6) (sc-137045), anti-mouse IgG-HRP (sc-2031), and anti-rabbit IgG-HRP (sc-2030) were purchased from Santa Cruz Biotechnology, Inc. Puromycin was purchased from InvivoGen (ant-pr-1). Growth factor reduced (GFR)-Matrigel^TM^ (CB-40230) was purchased from Corning. Lipofectamine 2000 (#11668027) and Plus reagent (#11514015) were purchased from Life Technologies. 4-hydroxytamoxifen (4-OHT) was purchased from Tocris Bioscience (#3412). SB203580 was purchased from Sellekchem (#S1076).

### Cell lines

MCF-7 was purchased from ATCC and maintained in DMEM media supplemented with 10% fetal bovine serum (FBS), 1% penicillin/streptomycin (PS), and 1× GlutaMAX. MCF-7, T47D, and all endocrine therapy-resistant derivative cells were developed and maintained as previously described.^32,33^ Briefly, T47D cells were maintained in RPMI-1640 media supplemented with 10% FBS, 1% PS, and 1× sodium pyruvate. MCF-7L-TamR and T47D-TamR cells were maintained in phenol red-free RPMI-1640 media supplemented with 10% Charcoal dextran-stripped FBS, 1% PSG, and sodium pyruvate. The TamR and FulvR cell lines were maintained with media containing charcoal-stripped serum in the presence of 100 nM 4-OHT and 100 nM of fulvestrant, respectively. For estrogen deprivation-resistant (EDR) cell lines, the cells were maintained in media containing charcoal-stripped serum as previously described.^32,33^ The absence of mycoplasma contamination was verified by Mycoalert PLUS mycoplasma detection kit (Lonza, #LT07-703).

### Viral infections

Lentivirus and retrovirus were generated as previously described.^25^ For stable cell generation, filtered retrovirus was incubated with target cells in 6-well plates overnight at 37 °C. Infected cells were selected with puromycin (2 µg/ml). For shRNA lentiviral infections, cells incubated with virus (1:1 ratio) overnight at 37 °C in the presence of polybrene (8 µg/ml).

### 3D cell growth assays

4 × 10^3^ of MCF-7L-TamR or T47D-TamR cells infected with control or PTK6 shRNA virus (C9 and 49) were seeded onto 8-well chamber slides coated with 50 μl of GFR-Matrigel^TM^. Cells were re-fed with complete growth media every 3–4 days and cells were imaged using the Axiovert 25 inverted microscope (Carl Zeiss AB). All 3D assays shown are representative of three independent experiments.

### Tumor xenograft studies

Six-week-old female nude (nu/nu) mice (Charles River Laboratories) were subcutaneously inserted with 17β-estradiol pellet (0.36 mg, 60-or-90-day release; Innovative Research of America; catalog #SE-121; NE-121) prior to tumor cell injection. MCF-7 or MCF-7L-TamR cells were infected with control or PTK6 shRNA lentivirus (C9 and 49). 66.6% GFR-Matrigel^TM^/media solution containing 3–5 × 10^6^ virus-infected cells were subcutaneously injected into the flanks of nude mice (*n* = 5/shRNA group). This number was required to detect 50% tumor growth difference between control and PTK6shRNA treatment groups with 82% power for a two-sided test at the 0.05 level. Cancer cells expressing PTK6 shRNA or vector control shRNA were generated in vitro prior to subcutaneous injection into nude mice. Mice from the combined purchased cages were randomly assigned to injection with PTK6 or control shRNA-expressing cells. Tumor growth was monitored twice weekly, and tumor volume was determined [V = 1/2(L x W^2^)]. Tumor measurements were performed by a second investigator who was blinded to the shRNA treatment. The mice were euthanized when the tumor diameters reached 10 mm in any direction according to approved IACUC protocol. All procedures and studies with mice were performed in accordance with protocols approved by the Institutional Animal Care and Use Committee of Icahn School of Medicine at Mount Sinai.

### Survival analysis

Age-corrected, batch-corrected, race-corrected, and gender-corrected and quantile-normalized TCGA RNAseqV2 data were used to assess association of PTK6 expression with survival in ER+ breast cancer samples. Samples were split into roughly equal tertiles based on log2-transformed expression. The upper and lower tertiles were designated as “high” and “low” expression groups, respectively, and association with survival was computed using the Survival R package. The log2-transformed expression values for PTK6 were 6.88 in “low” (*n* = 207) and 9.26 in “high” (*n* = 207), for a fold change of 2^(9.26-6.88)^ = 5.22. Statistical significance was assessed using a log rank test. The V2 RNAseq data from TCGA is not normalized to an internal control, but rather for total reads for a given sample that aligned to the transcriptome as well as for transcript length.

### Growth curve analysis

T47D cells stably expressing either pBabe vector control or MF-PTK6 were incubated in 5% CS-FBS containing phenol red-free RPMI-1640 media for 3 days. 3 × 10^4^ cells were seeded in 24-well plates in triplicate (technical replicates) and cells were re-fed with media containing either vehicle control or 100 nM of 4-OHT every other day. Cells were counted every 4–5 days to generate the growth curve. 1 × 10^4^ MCF-7 cells stably expressing either pBabe or MF-PTK6 were seeded in 24-well plate in triplicates (technical replicates) and cells were re-fed with complete growth media (DMEM with 10% FBS) or phenol red-free DMEM media containing vehicle control or 1 µM of 4-OHT every other day. Cells were counted every 2–3 days to generate growth curve. Experiments were performed three times.

### Apoptosis assay

Culture medium containing floating cells was transferred to 15 ml canonical tubes. Attached cells were collected by trypsinization at 37 °C in the same tubes followed by spinning at 360 × *g* for 3 min. Cell pellets were washed with ice-cold PBS once and resuspended in 250 µl of 1× binding buffer containing 5 μl of FITC-AnnexinV and PI (BD Biosciences, #556547). After 15 min incubation at room temperature in the dark, 250 µl of 1× binding buffer was added and flow cytometry analysis was performed.

### Western blot analysis

Cells were lysed in 1% NP-40 lysis buffer containing NaF, Na_3_VO_4_, Leupeptin, PMSF, aprotinin, and phosphatase inhibitor (PhosSTOP, Roche) as described previously.^25^ Cleared cell lysates were stored at −80 °C and were analyzed by western analysis as described previously.^25^

### p38 MAP kinase assay

Cells were lysed in 360 µl of lysis buffer following the manufacture’s protocol (Cell Signaling Technology, #9820), sonicated for 15 s for six times in a sonicator bath (Branson Ultrasonic bath, M1800H) followed by spinning at 20,000 × *g* for 10 min. Total protein concentration was measured using BCA assay and cell extract containing 500 µg of protein was incubated with 10 µl of phospho-p38 antibody conjugated beads slurry overnight at 4 °C. Immunoprecipitated active p38 was subjected to kinase reaction following the manufacture’s protocol. Kinase reaction was terminated by adding 4× SDS sample buffer and boiled at 95 °C for 5 min.

### Statistical analysis

GraphPad Prism v6.0d was used to perform all statistical analyses. Data were shown as mean ± standard error of mean from three independent experiments. Normal distribution was first determined by D’Agostino-Pearson omnibus normality test. When normal distribution was indicated, unpaired Student’s *t-*test (two-tailed) was performed to calculate significant differences between control and experimental groups considered significant (*P < 0.05, **P < 0.01, ***P < 0.001). *F-*test was also performed to confirm similar variances of groups. For the tumor xenograft experiments or data failing the normality test, Mann–Whitney non-parametric test was used.

### Data availability

RNA-seq data that support the findings of this study are available from The Cancer Genome Atlas (TCGA), downloaded from the UCSC Xena Browser (http://xena.ucsc.edu).

## Electronic supplementary material

Scanned unprocessed blots

Supplemental Figure 1
