# Supplementary material for: PTK6 regulates growth and survival of endocrine therapy-resistant ER+ breast cancer cells
Source: NPJ Breast Cancer. 2017 Nov 17;3:45. doi: 10.1038/s41523-017-0047-1 (PMC5694002; doi:10.1038/s41523-017-0047-1)

Un-processed WB blots

**Fig.1b: MCF7 (pBabe/MF-PTK6)**

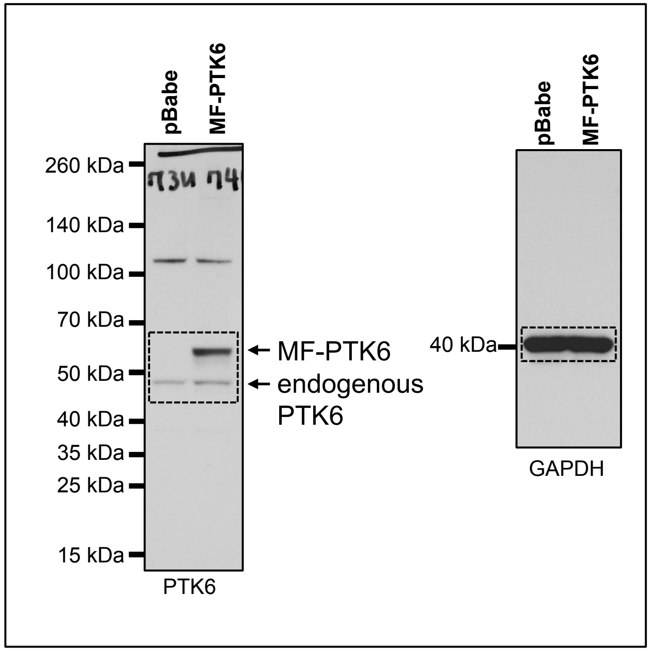


**Fig.1c: T47D (pBabe/MF-PTK6)**

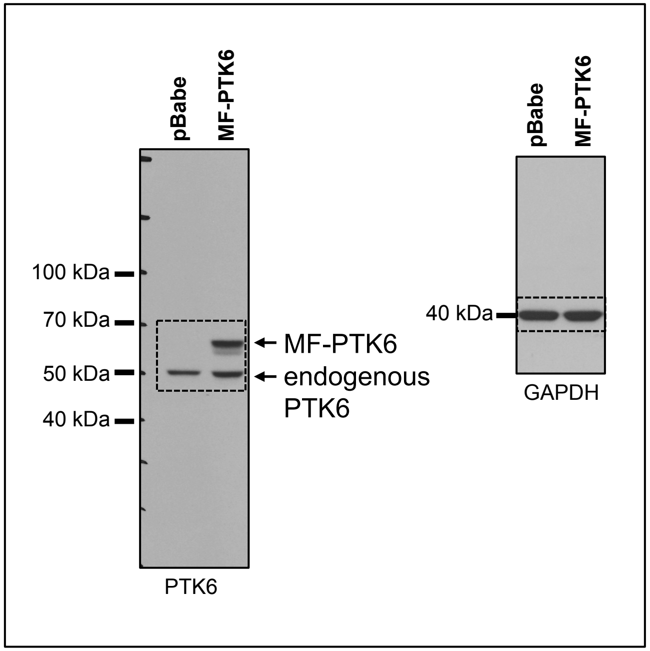

Fig2.

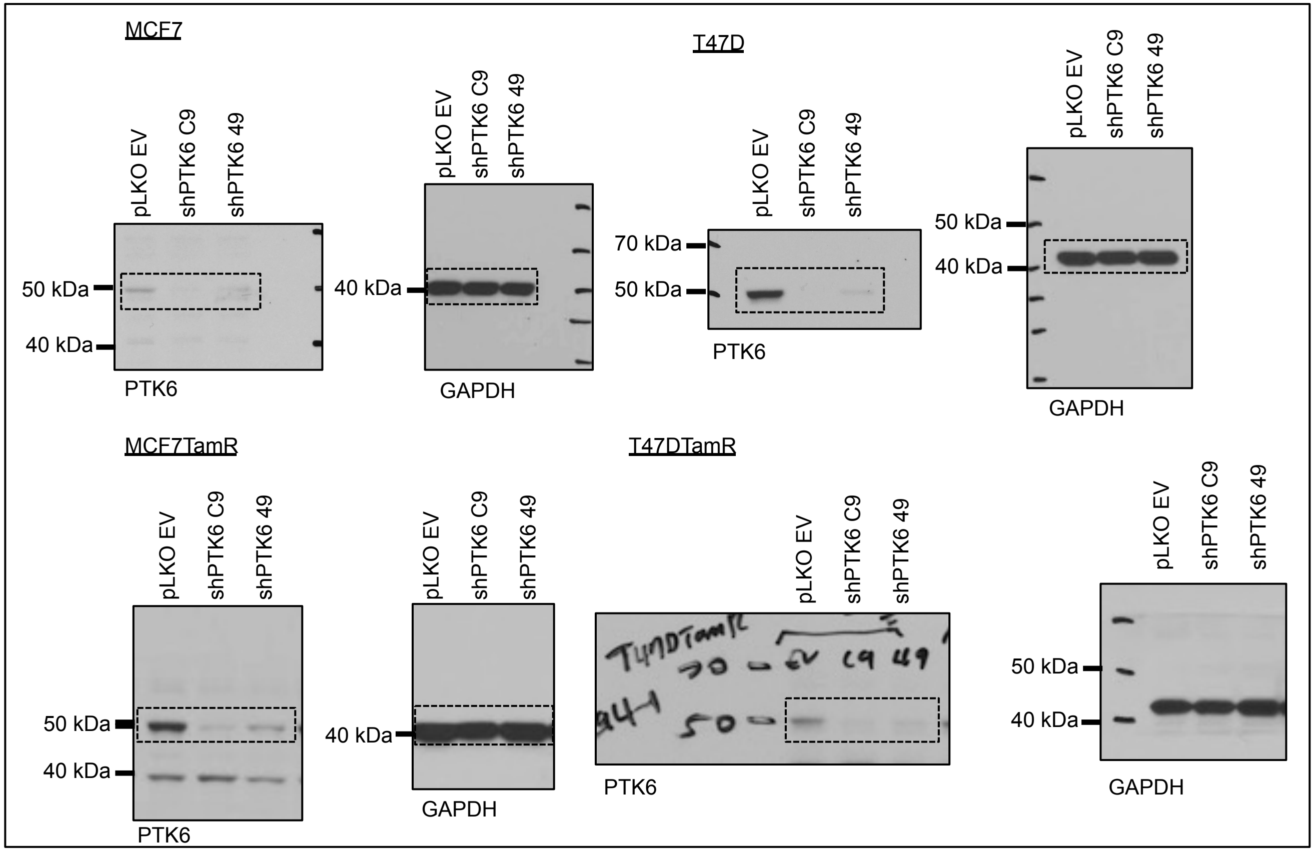
MCF7
pLKO EV
shPTK6 C9
shPTK6 49
50 kDa
40 kDa
PTK6
40 kDa
GAPDH
T47D
70 kDa
50 kDa
PTK6
50 kDa
40 kDa
GAPDH
MCF7TamR
50 kDa
40 kDa
PTK6
40 kDa
GAPDH
T47DTamR
PTK6
50 kDa
40 kDa
GAPDH

**Fig. 3a**

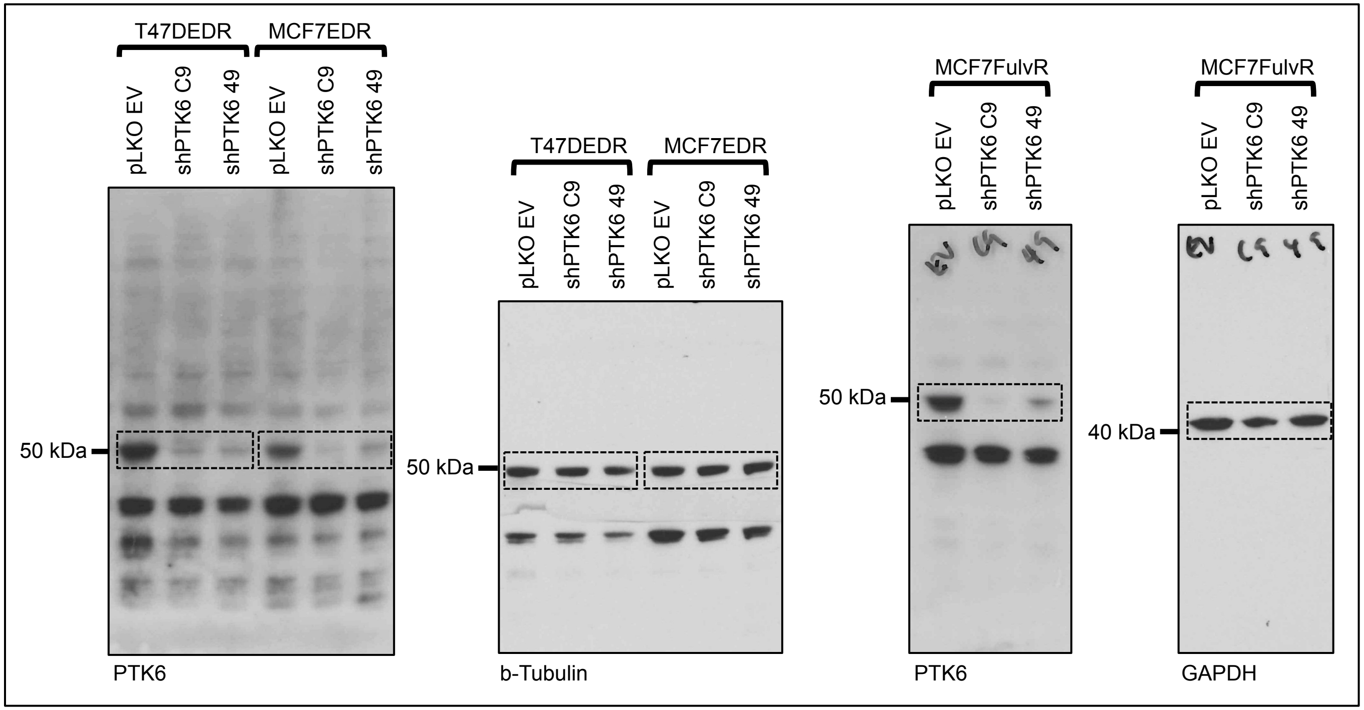
T47DEDR
MCF7EDR
pLKO EV
shPTK6 C9
shPTK6 49
50 kDa
PTK6
b-Tubulin
MCF7FulvR
40 kDa
GAPDH

Fig.4a.

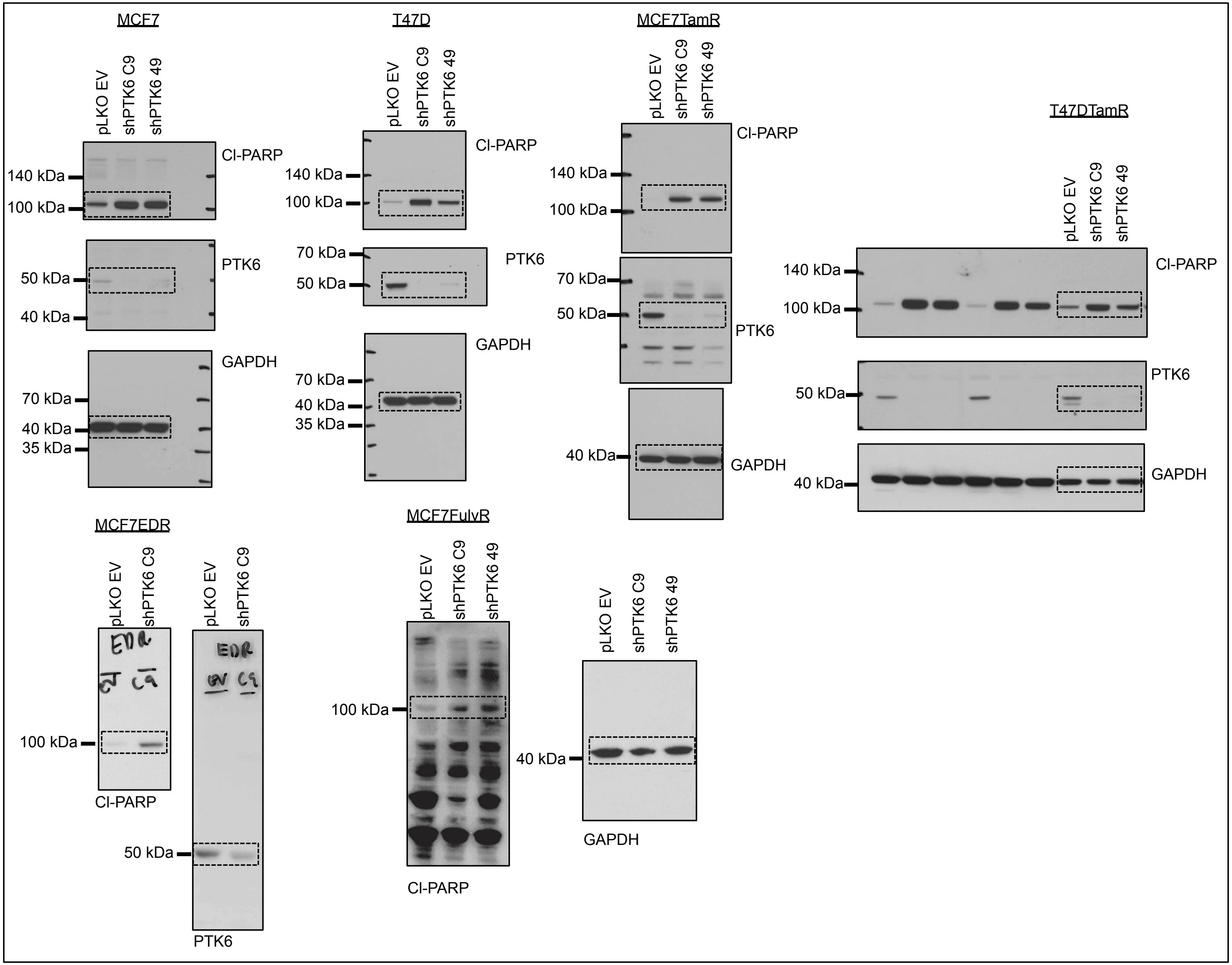
MCF7
pLKO EV
shPTK6 C9
shPTK6 49
Cl-PARP
140 kDa
100 kDa
PTK6
50 kDa
40 kDa
GAPDH
70 kDa
40 kDa
35 kDa
T47D
pLKO EV
shPTK6 C9
shPTK6 49
Cl-PARP
140 kDa
100 kDa
PTK6
70 kDa
50 kDa
GAPDH
70 kDa
40 kDa
35 kDa
MCF7TamR
pLKO EV
shPTK6 C9
shPTK6 49
Cl-PARP
140 kDa
100 kDa
70 kDa
50 kDa
PTK6
40 kDa
GAPDH
T47DTamR
pLKO EV
shPTK6 C9
shPTK6 49
Cl-PARP
140 kDa
100 kDa
PTK6
50 kDa
GAPDH
40 kDa
MCF7EDR
pLKO EV
shPTK6 C9
pLKO EV
shPTK6 C9
100 kDa
Cl-PARP
50 kDa
PTK6
MCF7FulvR
pLKO EV
shPTK6 C9
shPTK6 49
100 kDa
Cl-PARP
pLKO EV
shPTK6 C9
shPTK6 49
40 kDa
GAPDH

**Fig.5a**

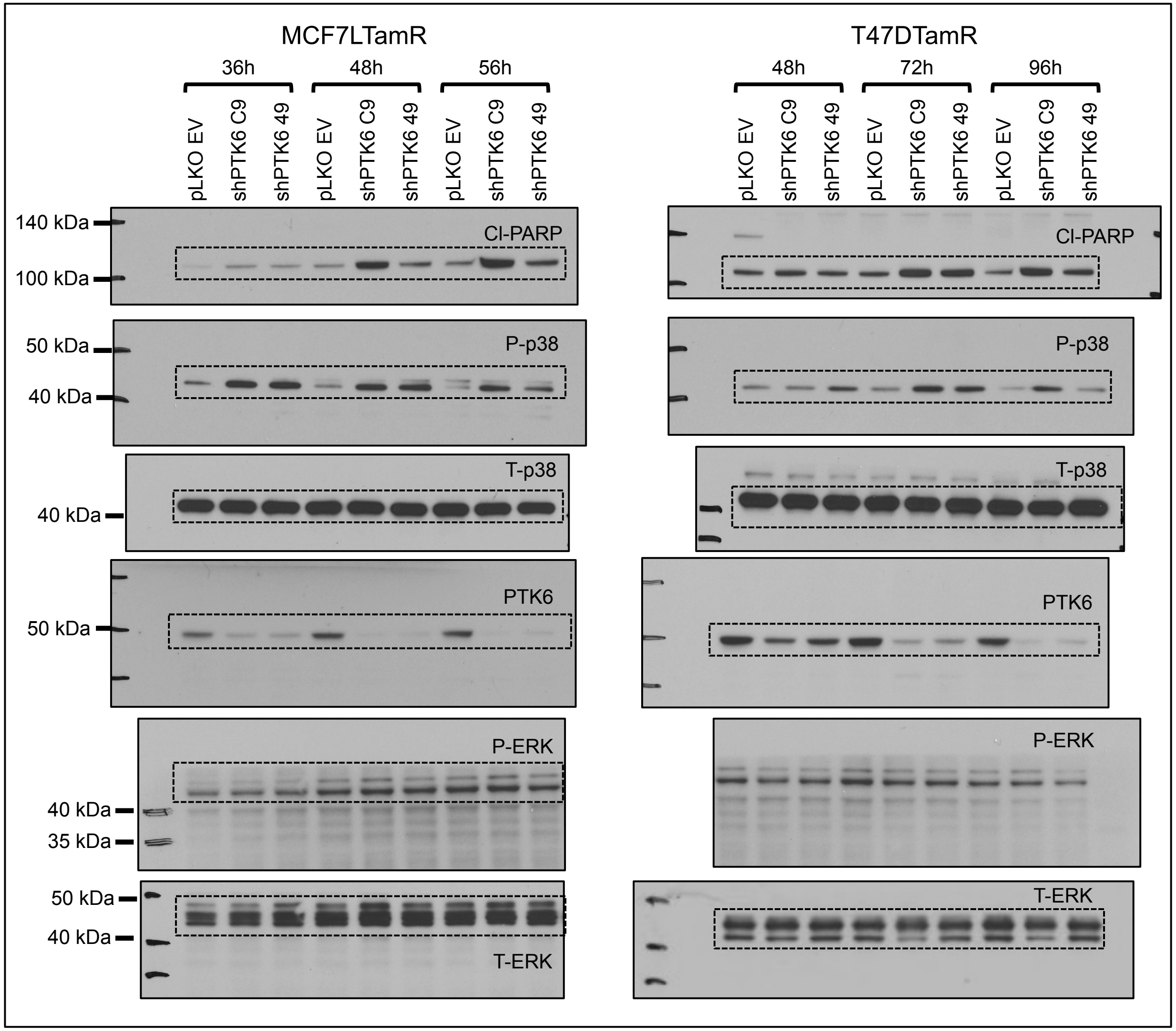
MCF7LTamR
T47DTamR
36h
48h
56h
48h
72h
96h
pLKO EV
shPTK6 C9
shPTK6 49
140 kDa
100 kDa
Cl-PARP
50 kDa
40 kDa
P-p38
T-p38
PTK6
P-ERK
35 kDa
T-ERK

**Fig.5a**

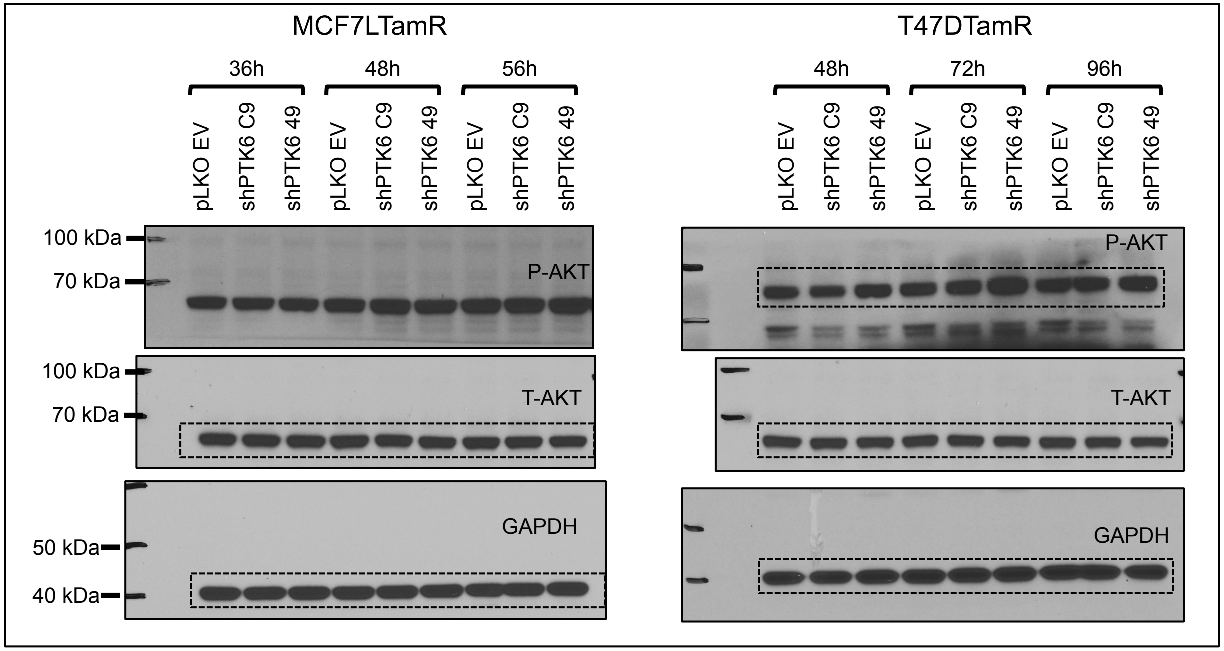


**Fig. 5b: MCF7LTamR**

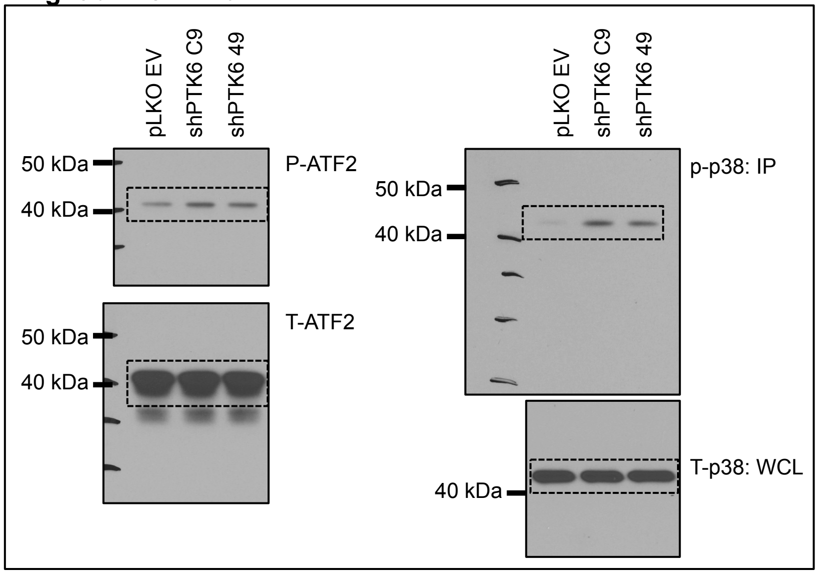

Fig.5C: MCF7TamR

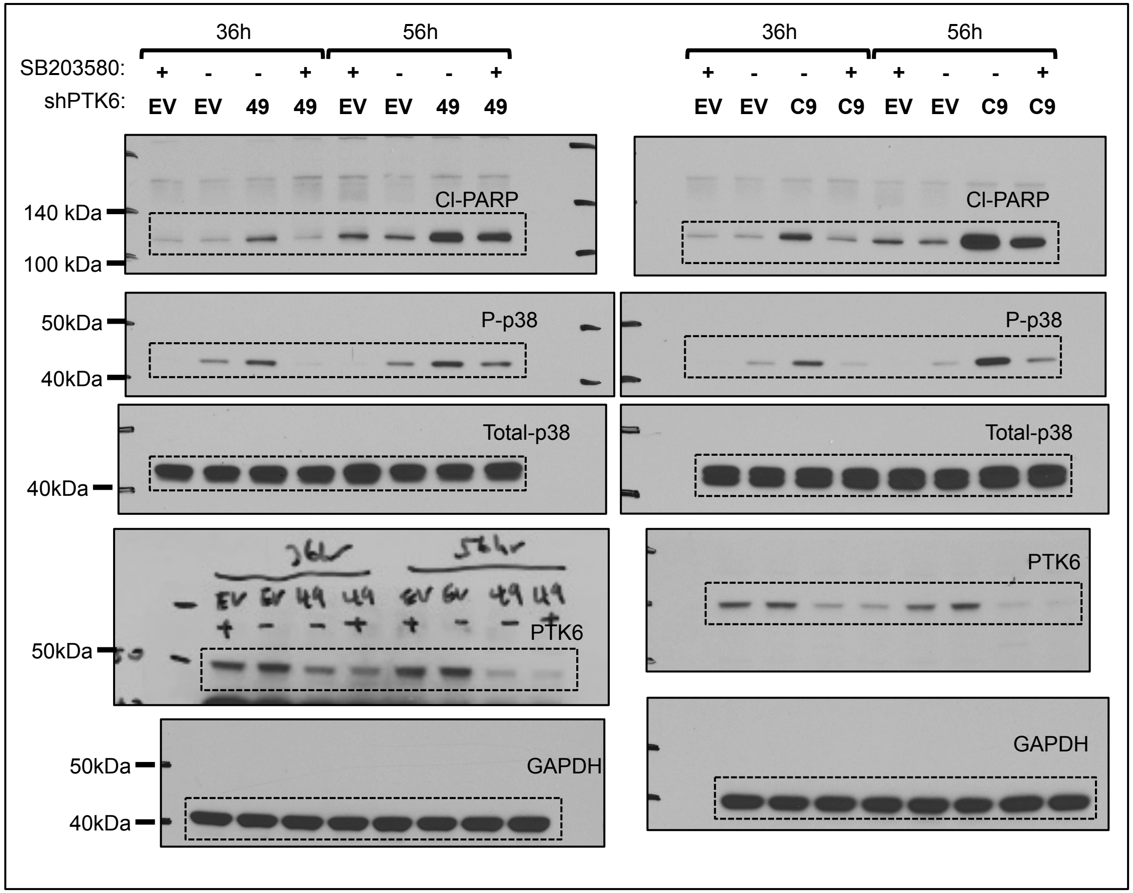


Fig.5C: T47DTamR

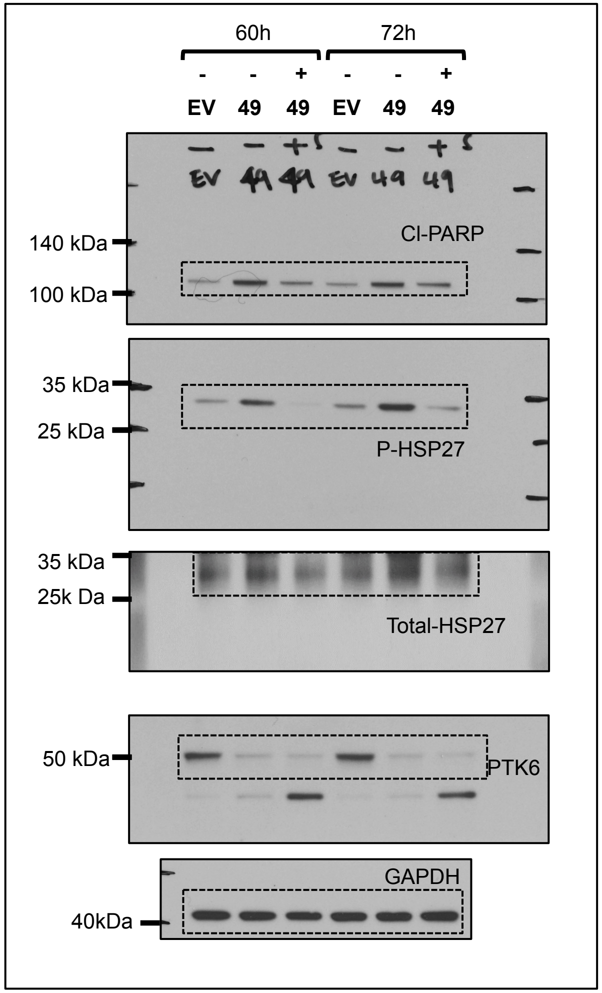

Supplement: Supplementary file 1 — Scanned unprocessed blots [file 41523_2017_47_MOESM1_ESM.pdf]
