# Supplementary material for: PTK6 regulates growth and survival of endocrine therapy-resistant ER+ breast cancer cells
Source: NPJ Breast Cancer. 2017 Nov 17;3:45. doi: 10.1038/s41523-017-0047-1 (PMC5694002; doi:10.1038/s41523-017-0047-1)
Supplement: Supplementary file 2 — Supplemental Figure 1 [file 41523_2017_47_MOESM2_ESM.pdf]

Supplemental Figure 1

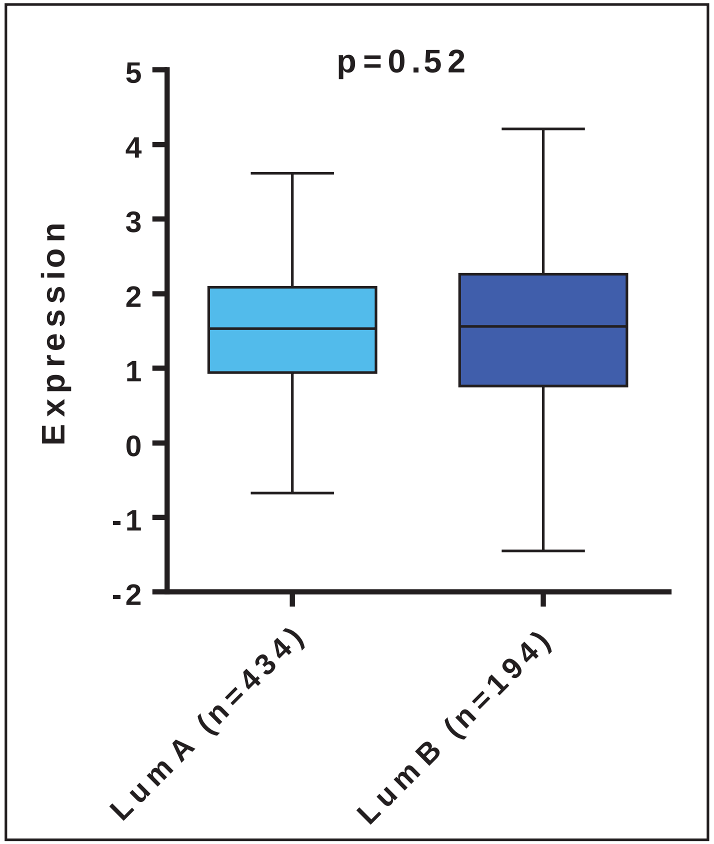

Supplementary Figure 1A. Boxplot showing PTK6 expression (RNA-seq, pan-cancer normalized log2(x+1) RSEM) in patients with Luminal A (LumA) and Luminal B (LumB) breast cancer molecular subtypes. RNA-seq data from The Cancer Genome Atlas (TCGA) were downloaded from the UCSC Xena Browser (http://xena.ucsc.edu).
